# From MEFs to Matrigel 3: Passaging hESCs from Matrigel onto Matrigel

**DOI:** 10.3791/832

**Published:** 2008-06-10

**Authors:** Jin Zhang, Ivan Khvorostov, Michael Teitell

**Affiliations:** David Geffen School of Medicine, University of California, Los Angeles

## Abstract

This video demonstrates how to maintain the growth of human embryonic stem cells (hESCs) in feeder cell-free conditions and how to continuously passage hESCs in feeder cell-free conditions. Confirmation of hESC pluripotency grown in feeder cell-free conditions by immunofluorescence microscopy is also demonstrated.

**Figure:**
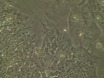


## Protocol

### Splitting hESCs from Matrigel to Matrigel

Usually a confluent 6-well plate of hESCs on Matrigel can be split 1:3 to 1:5 to another Matrigel plate, with the wells becoming confluent again 4-5 days after splitting.

1. CM and Matrigel plates are prepared as described above before splitting.
2. On the day of splitting, wash each well for splitting with 1×PBS, pH7.4, add 1ml of 1mg/ml Dispase (dilute 5mg/ml stock solution with DMEM/F12 media) to each well, and incubate at 37°C for 3 min.
3. Wash each well three times with 1×PBS, pH7.4, add 1ml of ES media without bFGF, use a cell scraper to scrape colonies off the bottom of the well, collect the suspended hESCs in a 50ml falcon tube, and pellet by centrifugation at 200g for 5 min at room temperature.
4. To re-plate the hESCs on Matrigel plates, first wash the Matrigel plates with DMEM/F12 and add 2.5ml of CM supplemented with 10ng/ml bFGF per well. Resuspend the hESC pellet in an appropriate volume of CM supplemented with 10ng/ml bFGF, pipette the cell suspension up and down three times. (NOTE: When hESCs for splitting come from Matrigel plates, the clumps are very easy to break; therefore three rounds of pipetting is enough and more pipetting may over-disrupt the colonies and make single cells.) Aliquot 0.5 ml of hESC clumps suspension per well of the 6-well Matrigel plate to achive 3ml per well as a final volume. Place the plate in a 37°C incubator.

### Detection of pluripotency markers by immunofluorescence microscopy

Immunofluorescence microscopy is used to examine the expression of hESC pluripotency markers Oct-4 and SSEA-4 during culturing.

1. Aspirate media from one well and wash with 1×PBS, pH 7.4. Add 2ml of 3.7% formaldehyde to the well to fix for 10 min.
2. Rinse with 1xPBS, pH 7.4, add 2ml methanol to the well and place at -20°C for 2 min.
3. Rinse with 1xPBS, pH 7.4, and block with 1ml of 0.2% bovine serume albumin (BSA) for 5 min.
4. Add 1ml of Oct-4 or anti-h/mSSEA-4 antibody (1:200 dilution in 0.2% BSA), and incubate at room temperature for 1 hr or at 4°C overnight. (NOTE: for SSEA-4 staining, go directly to step 6)
5. Rinse three times with 1xPBS, pH 7.4, add FITC-conjugated rabbit IgG (1:500 dilution in 0.2% BSA), and incubate at room temperature for 1 hr.
6. Rinse 3 times with 1xPBS, pH 7.4, and put a drop of mounting solution (can contain DAPI if nuclear visualization is desired) at the center of the well. Put coverslip on top of the cells, seal with nail polish, and evaluate by immunofluorescence microscopy.

### Human embryonic stem cell receipts

#### ES media (Embryonic Stem cell media):

DMEM/F12 - 400mlKnockout Serum Replacer - 100mlNon-Essential Amino Acids - 5ml200mM GlutaMax / BME Solution - 5mlPenicillin / Streptomycin - 5ml

Mix well and store final hESC Culture Media in 500ml bottles at 4°C. Good for a maximum of two weeks.

#### MEF Culture Media:

DMEM - 1000mlFBS - 100mlNon-Essential Amino Acids - 10mlGlutamine - 10mlPenicllin / Streptomycin - 10ml

Mix well and store final MEF Culture Media at 4°C.

#### 200mM Glutamax / BME Solution:

Glutamax - 100mlβ-mercaptoethanol (Stock solution at 14.3M) - 140 µ1Mix two solutions well and then aliquot 10ml portions. Keep aliquots at -20°C.bFGF Solution (final concentration of stock: 10 micrograms/ml):bFGF (FGF2) - 50 µg0.1% BSA in 1xPBS, pH 7.4 - 5ml

Dissolve 50 micrograms bFGF in 5ml 0.1% BSA to make a 10 µg/ml stock. Aliquot into 500 µl portions. Store at -80°C.

#### Collagenase IV Solution (1mg/ml):

Collagenase IV - 50mgDMEM/F12 Media - 50ml

Add the collagenase IV powder to a 50ml falcon tube. In the hood, mix in 50ml of sterile DMEM/F12 media. Vortex the solution for < 1 min. to make sure the powder is dissolved. In the hood, pass through a 0.22 µm filter into a new sterile 50ml falcon tube. This solution is good for a maximum of 2 weeks stored at 4°C.

#### Dispase Solution (1mg/ml):

Dispase 5mg/ml - 2mlDMEM/F12 Media - 8mlAliquot commercial 5mg/ml Dispase solution and store at -20°C. Dilute dispase to 1mg/ml using DMEM/F12. This solution can be kept for 1 week at 4°C.

## Discussion

This series of 3 videos demonstrates how to grow human embryonic stem cells (hESCs) on mouse embryonic fibroblast (MEF) feeder cells (video 1), how to passage them to Matrigel feeder cell-free plates (video 2), and how to maintain hESCs by passaging in Matrigel feeder cell-free conditions (video 3). Numerous prior studies showed that maintenance of viable, undifferentiated hESCs requires culture on inactivated MEF feeder cells. However, for many experiments, a pure population of hESCs free of feeder cell contamination is required. To achieve this goal, we have demonstrated how to passage hESCs from MEF feeder plates to feeder cell-free Matrigel plates and how to maintain and continuously passage hESC colonies in feeder cell-free conditions. By following this protocol, a small amount of MEF feeder cell contamination could still exist at passage one on a Matrigel plate, but this contaminant is usually easy to visualize. At Matrigel passage two and beyond, no MEF contamination is usually found, leaving a pure population of hESCs for experiments. Immunofluorescence staining and microscopy or flow cytometry for hESC pluripotency markers, such as Oct-4 and SSEA-4, are needed to confirm maintenance of hESCs in an undifferentiated state during culture.
